# A European multicentre evaluation of detection and typing methods for human enteroviruses and parechoviruses using RNA transcripts

**DOI:** 10.1002/jmv.25659

**Published:** 2020-01-17

**Authors:** A. Hayes, D. Nguyen, M. Andersson, A. Antón, J.‐L. Bailly, S. Beard, K. S. M. Benschop, N. Berginc, S. Blomqvist, E. Cunningham, D. Davis, J. L. Dembinski, S. Diedrich, S. G. Dudman, R. Dyrdak, G. J. A. Eltringham, S. Gonzales‐Goggia, R. Gunson, H. C. Howson‐Wells, A. J. Jääskeläinen, F. X. López‐Labrador, M. Maier, M. Majumdar, S. Midgley, A. Mirand, U. Morley, S. A. Nordbø, S. Oikarinen, H. Osman, A. Papa, L. Pellegrinelli, A. Piralla, N. Rabella, J. Richter, M. Smith, A. Söderlund Strand, K. Templeton, B. Vipond, T. Vuorinen, C. Williams, E. Wollants, K. Zakikhany, T. K. Fischer, H. Harvala, P. Simmonds

**Affiliations:** ^1^ Nuffield Department of Medicine University of Oxford Oxford UK; ^2^ Microbiology Laboratory, John Radcliffe Hospital, Headley Way, Headington Oxford UK; ^3^ Respiratory Viruses Unit, Virology Section, Microbiology Department Hospital Universitari Vall d'Hebron, Vall d'Hebron Research Institute, Universitat Autònoma de Barcelona, Passeig Vall d'Hebron Barcelona Spain; ^4^ Université Clermont Auvergne, LMGE UMR CNRS, UFR Médecine Clermont‐Ferrand France; ^5^ CHU Clermont‐Ferrand, National Reference Center for EV and Parechovirus‐Associated Laboratory Clermont‐Ferrand France; ^6^ Enteric Virus Unit, Virus Reference Department National Infection Service, Public Health England London UK; ^7^ National Institute for Public Health and the Environment (RIVM) Bilthoven The Netherlands; ^8^ Department for Public Health Virology National Laboratory of Health, Environment and Food Ljubljana Slovenia; ^9^ National Institute for Health and Welfare, Mannerheimintie Helsinki Finland; ^10^ Viapath Infection Sciences, St. Thomas' Hospital London UK; ^11^ Microbiology, Virology and infection Prevention & Control Great Ormond Street Hospital for Children NHS Foundation Trust London UK; ^12^ Department of Virology Norwegian Institute of Public Health Oslo Norway; ^13^ National Reference Center for Poliomyelitis and Enteroviruses, Robert Koch Institute Berlin Germany; ^14^ Department of Microbiology Oslo University Hospital Rikshospitalet, Inst. Clinical Medicine, University of Oslo Oslo Norway; ^15^ Department of Clinical Microbiology Karolinska University Hospital Stockholm Sweden; ^16^ Department of Microbiology, Tumor and Cell Biology Karolinska Institute Stockholm Sweden; ^17^ Molecular Diagnostics Laboratory, Microbiology, Freeman Hospital Newcastle Upon Tyne UK; ^18^ Public Health England Poliovirus Reference Laboratory, National Infection Service, Public Health England London UK; ^19^ West of Scotland Specialist Virology Centre Glasgow Royal Infirmary Glasgow UK; ^20^ Nottingham University Hospitals NHS Trust, Clinical Microbiology, Queens Medical Centre Nottingham UK; ^21^ University of Helsinki and Helsinki University Hospital, HUSLAB, Virology and Immunology Helsinki Finland; ^22^ Virology Laboratory, Joint Units in Genomics and Health and Infection and Health, Fundación para el Fomento de la Investigación Sanitaria y Biomédica de la Comunidad Valenciana (FISABIO‐Public Health)/Universitat de València, Av. Catalunya València Spain; ^23^ CIBEResp, Centro de Investigación Biomédica en Red en Epidemiología y Salud Pública, Instituto de Salud Carlos III Madrid Spain; ^24^ Institute of Virology Leipzig University Hospital Leipzig Germany; ^25^ The National Institute for Biological Standards and Control Hertfordshire UK; ^26^ Department of Virus and Special Microbiological Diagnostics Virus Surveillance and Research Section, Statens Serum Institut Copenhagen Denmark; ^27^ CHU Clermont‐Ferrand, Laboratoire de Virologie—Centre National de Référence des Entérovirus et Parechovirus, Laboratoire Associé—Clermont‐Ferrand France; ^28^ UCD National Virus Reference Laboratory University College Dublin, Belfield Dublin Ireland; ^29^ Department of Medical Microbiology St. Olavs University Hospital Trondheim Norway; ^30^ Department of Clinical and Molecular Medicine, Faculty of Medicine and Health Sciences Norwegian University of Science and Technology Trondheim Norway; ^31^ Faculty of Medicine and Health Technology Tampere University Tampere Finland; ^32^ Public Health England Birmingham Public Health Laboratory, Heartlands Hospital Birmingham UK; ^33^ Department of Microbiology Medical School, Aristotle University of Thessaloniki Thessaloniki Greece; ^34^ Department of Biomedical Sciences for Health University of Milan Milan Italy; ^35^ Molecular Virology Unit, Microbiology and Virology Department Fondazione IRCCS Policlinico San Matteo Pavia Italy; ^36^ Virology Section, Santa Creu i Sant Pau University Hospital Barcelona Spain; ^37^ Department of Molecular Virology Cyprus Institute of Neurology and Genetics Nicosia Cyprus; ^38^ King's College Hospital, Bessemer Wing, Denmark Hill London UK; ^39^ Laboratory Medicine, Department of Clinical Microbiology Lund University Hospital, Sölvegatan Lund Sweden; ^40^ Edinburgh Specialist Virology, Royal Infirmary of Edinburgh Edinburgh UK; ^41^ Public Health England, South West Regional Laboratory, Pathology Sciences Building, Science Quarter Southmead Hospital Bristol UK; ^42^ Clinical Microbiology Turku University Hospital and Institute of Biomedicine University of Turku Turku Finland; ^43^ Microbiology, Royal Oldham Hospital Oldham UK; ^44^ Clinical and Epidemiological Virology, KU Leuven, REGA Institute, Clinical and Epidemiological Virology Leuven Belgium; ^45^ Katherina Zakikhany‐Gilg, Public Health Agency of Sweden, Department of Microbiology Unit of Laboratory Surveillance of Viral Pathogens and Vaccine Preventable Diseases Stockholm Sweden; ^46^ NHS Blood and Transplant, Colindale London UK

**Keywords:** enterovirus, enterovirus A71, parechovirus, PCR, RNA transcripts

## Abstract

Polymerase chain reaction (PCR) detection has become the gold standard for diagnosis and typing of enterovirus (EV) and human parechovirus (HPeV) infections. Its effectiveness depends critically on using the appropriate sample types and high assay sensitivity as viral loads in cerebrospinal fluid samples from meningitis and sepsis clinical presentation can be extremely low. This study evaluated the sensitivity and specificity of currently used commercial and in‐house diagnostic and typing assays. Accurately quantified RNA transcript controls were distributed to 27 diagnostic and 12 reference laboratories in 17 European countries for blinded testing. Transcripts represented the four human EV species (EV‐A71, echovirus 30, coxsackie A virus 21, and EV‐D68), HPeV3, and specificity controls. Reported results from 48 in‐house and 15 commercial assays showed 98% detection frequencies of high copy (1000 RNA copies/5 µL) transcripts. In‐house assays showed significantly greater detection frequencies of the low copy (10 copies/5 µL) EV and HPeV transcripts (81% and 86%, respectively) compared with commercial assays (56%, 50%; *P* = 7 × 10^−5^). EV‐specific PCRs showed low cross‐reactivity with human rhinovirus C (3 of 42 tests) and infrequent positivity in the negative control (2 of 63 tests). Most or all high copy EV and HPeV controls were successfully typed (88%, 100%) by reference laboratories, but showed reduced effectiveness for low copy controls (41%, 67%). Stabilized RNA transcripts provide an effective, logistically simple and inexpensive reagent for evaluation of diagnostic assay performance. The study provides reassurance of the performance of the many in‐house assay formats used across Europe. However, it identified often substantially reduced sensitivities of commercial assays often used as point‐of‐care tests.

## 1 INTRODUCTION

Enteroviruses (EVs) belonging to the family *Picornaviridae* are currently classified into 116 serologically distinct enterovirus types, which can be assigned into four genetically distinct species, HEV‐A to D^1^, ^2^ and over 250 human rhinovirus (HRV) types divided into three species (A‐C). Species B EV types including echoviruses and coxsackie B viruses (CBVs) are the most frequently identified viral causes of meningitis and other central nervous system (CNS)‐associated infections in western countries, while the species A serotype, EV‐A71 is an important cause of hand, foot, and mouth disease and encephalitis in South‐East Asia and EV‐D68 within species D has recently emerged as a respiratory pathogen occasionally leading to acute flaccid myelitis (AFM).^3^ Infections with human parechoviruses (HPeVs) in the genus *Parechovirus* are enteric, usually asymptomatic apart from those of HPeV type 3, which is associated with sepsis‐like illness, meningitis, and encephalitis in young children.^4^, ^5^, ^6^

Although there is no effective antiviral treatment available for EV infections, detection and identification of EV and HPeV infections are vital for informing other treatment options, supportive care and prognosis of affected individuals. The reverse‐transcriptase polymerase chain reaction (RT‐PCR) is now the “gold standard” for diagnosing EV and HPeV infections due to its advantages of fast turn‐around time and high sensitivity over virus isolation.^7^ Even in severe cases, viral loads are relatively low in cerebrospinal fluid (CSF) samples that are typically tested in patients presenting with meningitis or encephalitis, and may be missed by less sensitive methods.

To detect all EV types, RT‐PCR assays for the detection of EV RNA usually target the highly conserved 5′ non‐translated region (5′NTR). Depending on the primer and probe design, some molecular detection methods may fail to detect certain EV types such as EV‐D68, whereas some assays may also detect HRVs (reviewed in Holm‐Hansen^3^). EV and HPeV serotypes are defined serologically and genetically by their capsid region sequences; virus typing, therefore, requires amplification and sequencing of regions within this structural gene block, typically VP1.^8^, ^9^

Evaluation of sensitivity and specificity of the diagnostic assays used for EV and HPeV detection and typing is essential. We have previously evaluated the use of RNA transcripts of several EV and HRV serotypes and HPeV1 for quality control purposes in six expert clinical virology laboratories in Europe.^10^ Following this study, we have now produced a further set of RNA transcript standards for selected representative serotypes from EV species A‐D and HPeV3. The RNA standards were distributed via the European non‐polio enterovirus network (ENPEN) to members in diagnostic and reference laboratories for evaluation of the sensitivity of their routinely used assays for detection and typing of enteroviruses.

## 2 MATERIALS AND METHODS

### 2.1 RNA transcript synthesis

Available full‐length cDNA clones of EV species A (EV‐A71 genogroup B4 strain, accession number AF316321)^11^, B (Bastianni prototype strain of echovirus 30 [E30], AF162711), C (Coe strain of coxsackivirus A 21 [CVA21], D00538), and D (Fermon strain of EV‐D68, NC_038308), and parechovirus (HPeV3, GQ183026^12^) were selected for this study. For rhinovirus species C (HRV‐C49, MF775365), a partial 5′‐UTR‐VP4‐VP2 clone was assembled from amplified sequences. All plasmids were transformed into DH5α competent cells by heat shock, with single colonies picked and grown in liquid medium before plasmid extraction using the QIAprep Spin Miniprep Kit (Qiagen), according to the manufacturer's instructions. Plasmids were linearized at the 3′ end, and RNA transcripts were produced using the MEGAscript T7 Transcription Kit (Ambion), followed by DNase treatment to remove template DNA. RNA was purified using the RNAEasy Mini Kit (Qiagen), according to the manufacturer's instructions.

### 2.2 RNA quantification and stability assessment

Quantification of RNA transcripts was carried out using the NanoDrop ND‐1000 UV‐Vis Spectrophotometer (Thermo Fisher Scientific) and the Qubit 4 Fluorometer (Thermo Fisher Scientific). The concentrations obtained were used to calculate copy numbers of transcripts produced, assuming a mean molecular mass for each base of 330 g/mol. A serial dilution of RNA transcripts (10^5^ to 10^−1^ copies/µL) was prepared using the RNA storage solution (Thermo Fisher Scientific; 1 mM sodium citrate, pH 6.4) containing herring sperm carrier RNA (50 µg/mL) and RNasin (New England BioLabs UK, 100 U/mL). Dilutions were aliquoted and stored at −80°C before testing and distribution to the participating laboratories.

EV species A (EV‐A71) and C (CVA21) transcripts were investigated for stability at different temperatures. Transcripts were incubated in storage solution for up to 30 days at ambient temperature, 4°C and 37°C. A further aliquot of each was freeze‐thawed three times. The amount of RNA was quantified by RT‐PCR and values compared to those of the original preparations.

### 2.3 Transcript amplification by real‐time RT‐PCR

For quantification of RNA sequences before distribution, In‐house quantitative real‐time RT‐PCR was carried out using the StepOnePlus Real‐Time PCR System (Thermo Fisher Scientific), according to the manufacturer's instructions. The following reaction conditions were used: 50°C for 30 minutes, 95°C for 15 minutes followed by 45 cycles of 95°C for 10 seconds then 60°C for 1 minute. A total of 20 μL reaction volume containing 2 μL of the diluted transcript was used. PCRs used primers and probes as previously described for EV,^13^ HRV,^14^ and HPeV^15^ (Table S1).

### 2.4 Laboratory evaluation

RNA from EV species A‐D and HPeV was distributed in 200 μL volumes of storage buffer at concentrations of 10 and 10^3^ copies/5 μL. The HRV species C, as a negative transcript control, was distributed only at the higher concentration (10^3^ copies in 5 μL) to investigate cross‐reactivity of EV assays with rhinoviruses. Storage buffer was included as another negative transcript control. Sample labeling was coded, and the details of coding were provided to participating laboratories only after all results were received. Coded transcript panels were sent by standard registered post to the participating laboratories in February 2019.

### 2.5 Participating laboratories

The RNA transcripts were distributed via ENPEN to member diagnostic and reference laboratories for evaluation of the sensitivity of their routinely used assays for detection and typing of EV and HPeV. Laboratories were identified by a standardized code (L1, L3….). Coded transcripts panels were shipped by standard registered UK post in February 2019. Participating labs were asked to test 5ul of each sample sent using their routine detection and/or typing assay. Results were reported through an EU survey tool (https://ec.europa.eu/eusurvey/runner/ENPEN_transcript_study_panel_A_2019). Collected information comprised the results of testing in terms of positivity or negativity, Ct values were obtained, virus type and further technical information such as volume tested, extraction method (if used) and additional information on the testing methods used (listed in Supplementary Methods).

## 3 RESULTS

### 3.1 Validation of the transcript panel

**Figure 1:**
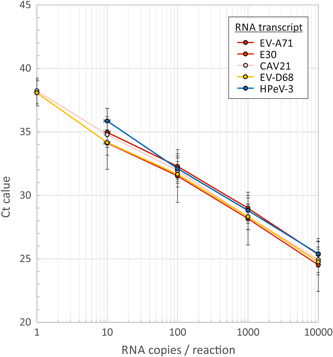
Quantitative RT‐PCR of RNA transcripts of different EV species, HRV and HPeV. *C*_t_ values of replicate dilutions of EV, HRV and HPeV RNA transcripts used in the evaluation panel; data points indicate mean values of 3 technical replicate; error bars show standard errors of the mean. EV, enterovirus; HPeV, human parechovirus; HRV, human rhinovirus; RT‐PCR, reverse‐transcriptase polymerase chain reaction

RNA concentrations of EV‐A71, E30, CVA21, EV‐D68, HPeV3, and HRV‐C49 transcripts were determined by two different physicochemical methods (Nanodrop and Qubit). The values obtained by the two methods' values were similar (within a factor of two in all cases; data not shown) and the mean value was used for calibration. Serial dilutions of each RNA transcript ranging from 10^4^ to 10^−1^ copies in 5 µL were assayed in replicate by quantitative EV and HPeV RT‐PCR assay. Amplification was highly reproducible between replicates and between transcripts of different EV species and HPeV3 (Figure 1). All samples were positive with an input of 10 copies, while detection of single copies of RNA was stochastic (2/5 replicates detected). The end‐point detection of the RNA transcripts and their similarity in *C*_t_ values to each demonstrates that quantitation was reproducible between the transcripts of the five different viral species (all *C*_t_ values were within a factor of two from each other), Finally, the measured gradients of the lines of best fit between log viral load and Ct‐value were between 3.1 and 3.6, consistent with efficient amplification.

**Figure 2:**
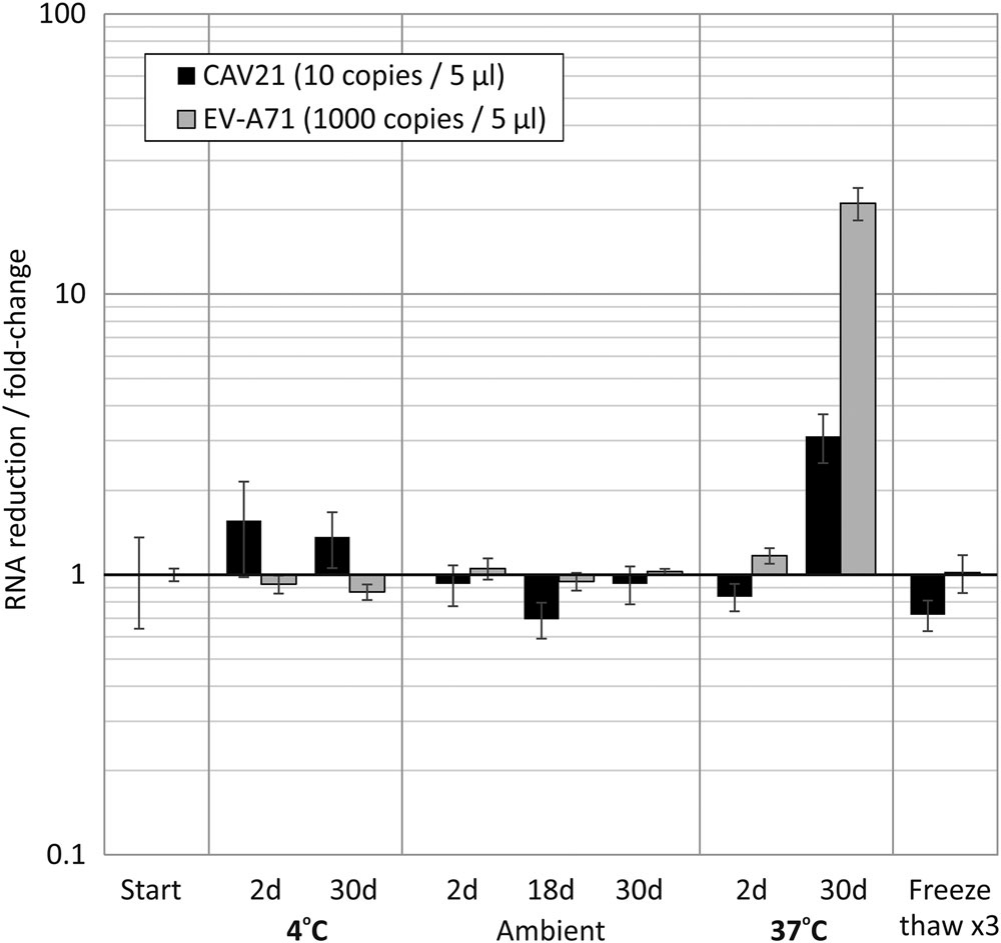
Stability of RNA transcripts on incubation at different temperatures and freeze/thawing. Fold changes in RNA detection of two representative RNA transcripts preparations of CAV21 (EV species C) at low copy number and EV‐A71 (species A, high copy number) used for laboratory distribution. Transcripts were incubated for various durations at different temperatures. Detected viral loads were compared to those of RNA transcripts stored at −80°C. RNA transcripts were additionally subjected to three freeze/thaw cycles (rapid cooling and thawing; right hand panel). Bar heights show fold reductions of RNA relative to the starting amount; error bars show SEMS of three assay repeats

To investigate the stability of RNA transcript, two representative EV transcripts (EV‐A71 and CVA21) were subjected to a range of temperatures and freeze‐thaw cycles and their RNA content was assessed by real‐time PCR (Figure 2). No or minimal changes in *C*_t_ values (reflecting residual RNA concentrations) were observed on freezing‐thawing or incubation for up to 30 days at 4°C or ambient temperate, while there was an approximately 10‐fold reduction in RNA levels on incubation at 37°C for 30 days No decline in *C*_t_ values was observed for any transcript stored at 4°C or room temperate over the month period, providing reassurance that transcripts received by the participating laboratories were not degraded.

A panel of 12 RNA transcripts was constructed containing two concentrations of EV‐A71, E30, CVA21, EV‐D68, and HPeV3 (10 and 1000 copies in 5 µL), HRV‐C49 (1000 copies in 5 µL) and a water control. These panels were sent to the participating laboratories using standard registered post at ambient temperature; delivery times ranged from one to 4 weeks providing reassurance for the RNA quality.

### 3.2 Participating laboratories

A total of 39 laboratories from 17 European countries including Belgium (number of participating laboratories = 1), Cyprus (1), Denmark (1), France (1), Finland (4), Germany (2), Greece (1), Italy (2), Ireland (1), Netherlands (1), Norway (3), Slovenia (1), Spain (3), Sweden (3), and UK (14) participated to this study. From these, 12 were classified as national reference laboratories and the remaining 27 as primary diagnostic laboratories (Table S2).

Most laboratories (*n* = 36) participated in the evaluation of detection assays, some evaluating multiple assays; this produced a total of 63 sets of results for detection assays. Reference Laboratories showed greater investment in in‐house detection methods for EV and HPeV detection; in‐house assays were used for 19 of the 22 results sets provided by 12 reference laboratories for the study (86%), with only one reference laboratory located within a local hospital contributing results from three different commercial assays). This compares 29/41 (71%) of in‐house result sets provided by 27 diagnostic laboratories. Commercial kits used included assays from BioMérieux, Seegene, Progenie, Luminex, Fast Track Diagnostics, Elite Ingenius, Biofire, Altona, and AusDiagnostics (Table 1).

**Table 1:** Testing results from individual commercial assay platforms

			Detection^1^		
Manufacturer	Assay	*C*_t_	10	1000	HRV‐C
Enterovirus assays					
Altona	RealStar	Y	4/4	4/4	**P**
BioMerieux	Enterovirus R	Y	4/4	4/4	N
EV/HPeV assays					
Ausdiagnostics	Resp. viruses 16‐well	N	0/5	**4/5** ^2^	N
Ausdiagnostics	Viral 8‐well version 3.0	N	2/5	5/5	N
Ausdiagnostics	Viral 8‐well version 01	N	1/5	**4/5** ^2^	N
Biofire	Film array ME panel v1.4	N	0/5	**4/5** ^3^	N
Elite‐Ingenious	Meningitis viral 2 MGB panel	Y	4/5	5/5	N
Fast Track	FTD Viral Meningitis	Y	5/5	5/5	N
Fast Track	FTD Neuro 9	Y	5/5	5/5	N
Luminex	NxTAG Resp. Pathogen Panel	Y	5/5	5/5	N
Progenie	Real Cycler EVPA ‐ Version 4	Y	2/5	4/4	N
Progenie	Real Cycler Monotest	Y	1/5	5/5	N
HRV/RV assays					
BioMerieux	Rhino/Entero R gene	Y	1/3	‐	P
Seegene	Allplex Resp. Panel 2‐ RP9802x	Y	1/4	3/3	**N**
Seegene	Allplex Resp. Panel RV16	Y	3/4	4/4	P
Parechovirus assays					
BioMerieux	Parechovirus r‐gene	Y	1/1	1/1	N

^1^ Detection frequencies in the 10 and 1000 copies/5 µL transcript dilutions. Insensitive results—low detection rate of the 10 copy/5 µL control—are underlined, unexpected results—detection failure of 1000 copy/5 µL controls are indicated in bold.

^2^ The 1000 copy/5 µL E30 (EV species B) transcript was undetected in both assays.

^3^ The 1000 copy/5 µL HPeV3 transcript was undetected.

A total of 37 sets of in‐house typing results were provided, from these 18 sets (14 for EV typing, 3 for HPeV, and 1 for both) were reported by reference laboratories and 12 sets by diagnostic laboratories from 7 different countries including Spain, Sweden, Greece, Italy, Germany, Norway, and UK. Most of these results were reported for EV typing (*n* = 29) but HPeV typing was also performed (*n* = 10).

### 3.3 Sensitivity and specificity of screening methods

The sensitivity and specificity of detection assay results were calculated, and totals adjusted for the declared target range of the tests. Intended assay targets included combined EV and HPeV detection (*n* = 23), EV detection only (*n* = 17), HPeV detection only (*n* = 10) and combined EV and HRV detection (*n* = 7) as well as mono‐specific assays for EV‐D68 (*n* = 1) and EV‐A71 (*n* = 1). Two HRV‐only assays were evaluated for specificity only.

**Figure 3:**
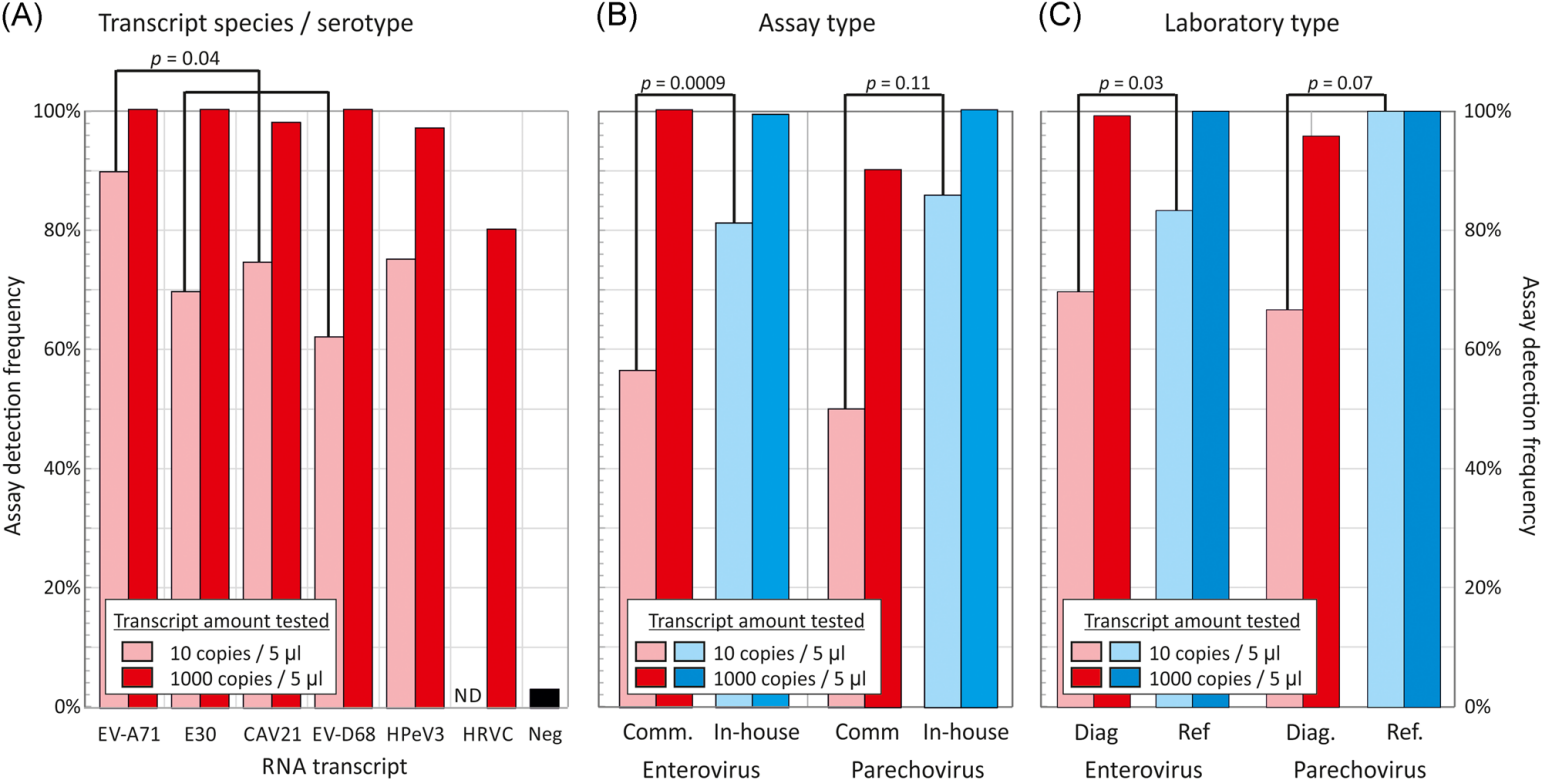
Detection frequencies of EV and HPeV transcripts. Frequencies of detection of the 10 and 1000 RNA copy/5 µL dilutions of each transcript by participant laboratories, divided by (A) transcript sequence, including detection frequencies of the HRV‐C and water negative controls. The detection frequency of the EV‐A71 transcript was significantly higher than achieved for the other EV species (B) Assay type—in‐house for commercially available (results for individual commercial assays are shown in Table 1), and (C) laboratory type, diagnostic or national reference laboratory both shown separately for the EV and HPeV transcripts. *P* values above bars show frequency comparisons using Fisher's Exact Test. EV, enterovirus; HPeV, human parechovirus; HRV‐C, human rhinovirus species C

In general, laboratories reported high rates of detection (98% for CVA21; 100% for EV‐A71, E30, and EV‐D68) of the EV transcripts at the higher concentration (10^3^ RNA copies in 5 µL) (Figure 3). More variable detection of the low concentration transcripts (10 RNA copies in 5 µL) was reported, ranging from 62% (EV‐D68) to 90% (EV‐A71). Detection frequencies of the HPeV3 transcripts were comparable; 97% for higher concentration and 75% for lower concentration. For assays reporting *C*_t_ values for the higher and lower concentration transcripts, values were compared to evaluate viral load ratios (Figure S1). Although no assay produced quantitative results, reported results showed a 58 to 106 fold differences in geometric mean viral loads, close to the expected 100‐fold difference. Assays were therefore reasonably quantitative in relative terms in this concentration range.

Assays were also generally highly specific, with only 2 high *C*_t_ value (weak positive) results reported falsely positive from the 62 tests performed. A larger number of tests specific for EVs reported HRV detection, with five tests designed for the detection of EV (*n* = 2), EV and HPeV (*n* = 2) and EV‐D68 (*n* = 1) reporting positive results with the HRV‐C49 RNA transcript.

Methodology differences contributed substantially to the sensitivity of the screening assays (Figure 3B). In particular, commercial assays, often highly multiplexed for other viral targets in CSF, showed significantly reduced sensitivity for the detection of RNA transcripts at a lower concentration compared to in‐house methods (56% detection rate compared to 81%; *P = *.0009; Figure 3B). There was a comparable difference in HPeV detection rates, with 50% of the lower concentration transcript detected by commercial and 86% by in‐house assays. There were significant differences in assay sensitivity in results reported by diagnostic and by reference laboratories (Figure 3C). This is largely accounted for by the greater use of in‐house assays by reference laboratories.

Commercial assays included a variety of platforms and assay specificities (Table 1); several including Biofire, AusDiagnostics, Progenie and some assays from Seegene and bioMérieux were unable to detect the lower concentration RNA control (10 RNA copies in 5 µL), and in some cases, even the higher concentration RNA transcript (1000 copies in 5 µL) (Biofire, AusDiagnostics). We further investigated the sensitivity of the Biofire assay with intermediate RNA concentrations; assay sensitivity lay between 400 and 1000 RNA copies for most of the EV transcripts and between 1000 and 40 000 copies for HPeV RNA (Table 2).

**Table 2:** Sensitivity of biofire film array assay

	RNA copies/5 µL			
Transcript	10	400	1000	40000
EV‐A71	N^1^	P^2^	P	P
E30	‐^3^	N	P	P
CAV21	‐	N	P	P
EV‐D68	‐	N	P	P
HPeV3	‐	N	N	P

^1^ Negative in assay.

^2^ Positive in assay.

^3^ Not done.

### 3.4 Sensitivity and accuracy of EV and HPeV typing methods

**Figure 4:**
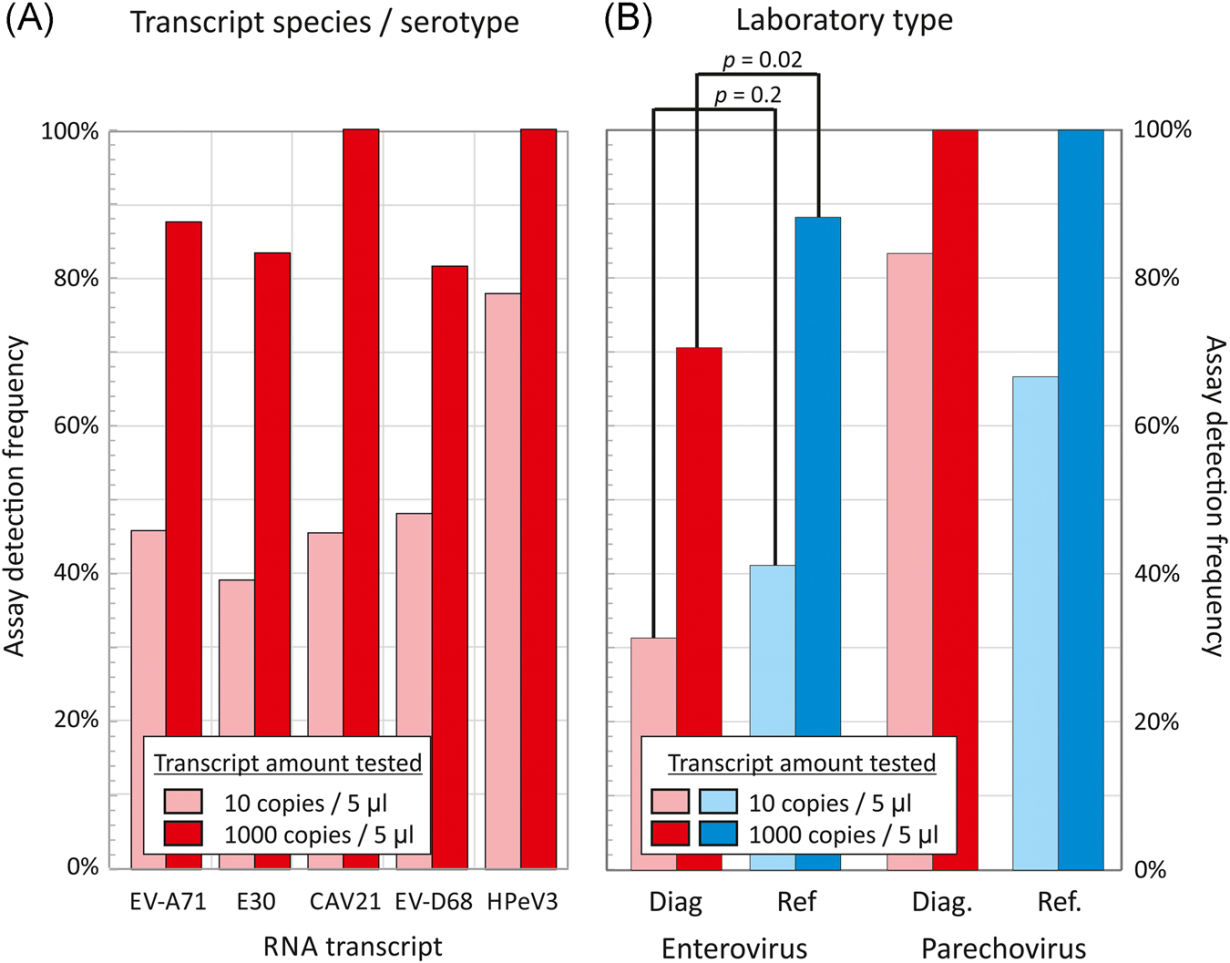
Frequencies of successful typing of EV and HPeV transcripts. Frequencies of successful typing of the 10 and 1000 RNA copy/5 µL dilutions of EV and HPeV transcripts divided by (A) transcript sequence and (B) laboratory type, diagnostic or national reference laboratory both shown separately for the EV and HPeV transcripts. *P* values above bars show frequency comparisons using Fisher's Exact Test. EV, enterovirus; HPeV, human parechovirus

EV and HPeV typing performed by 22 participating laboratories were based upon amplification by PCR and Sanger sequencing of VP1 and/or VP3/VP1 (EV) or VP3/VP1 (HPeV) regions either using species‐ or genus‐specific assays. Half of the typing was performed at the reference laboratories and half at the diagnostic laboratories resulting in the use of 37 different typing assays (Figure 4). The higher concentration EV RNA transcripts were successfully typed by 88% of reference laboratories (45 of 51) and 71% of diagnostic laboratories (36 of 57; *P = *.02 by Fisher's Exact Test) whereas the lower concentration EV transcripts were successfully typed by 41% of reference laboratories (21 of 51) and 31% of diagnostic laboratories (16 of 51; *P* = 0.2). High concentration HPeV RNA transcripts were successfully typed by all reference (3 of 3) and diagnostic laboratories (6 of 6) but one of each failed detection of the low copy HPeV transcript.

## 4 DISCUSSION

This study describes a quality control exercise for EV and HPeV detection and typing in 43 virus diagnostic and reference laboratories in 17 European countries. Intrinsic to the study design was the use of stabilized RNA transcripts of representative serotypes and species of EV and HPeV.

### 4.1 The suitability of RNA transcripts for quality assurance

RNA transcripts were used to evaluate the sensitivity and specificity of commercial and in‐house detection and characterization assays for EVs and HPeV. Multiple EV types were used in the panel to estimate the range of serotypes and species that could be detected; enteroviruses are substantially diverse genetically, and conserved regions of the genome between EV species suitable for amplification by PCR are largely confined to short motifs in the 5′‐UTR.^13^ EV and HPeV RNA transcripts possess a number of attributes that make them suitable as reagents for quality assurance (QA) and evaluation of diagnostic assays. Most importantly, their content can be quantified in absolute terms, so it is possible to express assay sensitivities in terms of RNA copies. This provides a stable quantitative standard for longer‐term evaluation of assays sensitivities. Secondly, the transcripts are based on uniform, defined sequences enabling assay failures to be investigated though comparisons of primer/probe and target sequences. Thirdly, unlimited amounts of RNA transcripts can be generated from cDNA clones of each target guaranteeing long term stability and reproducibility of the control materials. Fourthly, the RNA transcripts are highly stable at ambient temperature and on repeated freeze/thawing (Figure 2). Finally, they are noninfectious, enabling their international distribution by ordinary post, providing considerable cost savings, assured biological safety and simple logistics. RNA transcripts can indeed be very easily prepared on a large scale for little cost—in the current study, the consumable costs for laboratory production and packaging of the transcripts was approximately €120, while postage cost to the participating laboratories was approximately €390. This low cost enables participation in this and future RNA transcript‐based QA exercises to be uncharged. The wide availability and insignificant cost will facilitate the use of RNA transcripts in the development of screening and typing capacity in Eastern Europe, where EV detection is largely restricted to virus isolation and neutralization assays.^16^

There are however also some potential disadvantages to the use of RNA transcript controls and limitations of the study. Firstly, the RNA controls were directly used without RNA/DNA extraction in some test formats, so the efficiency of this step, which may be critical in clinical sample handling, and its downstream effects on overall assay performance was not evaluated for all assays. In practice, however, this can be evaluated through comparison of *C*_t_ values of transcript samples with and without an extraction step. The second potential disadvantage of RNA transcripts was the use of carrier RNA for stabilization. Although the concentrations used were relatively low in molecular terms (a 5 µL aliquot used for testing contained 250 ng of RNA), its presence could interfere with high throughput sequencing methods that may become increasingly used for virus typing in the future. However, the RNA amount used was well within the capacity of current library preparation methods for Illumina and Nanopore methods, and indeed cDNA in these concentrations may be required for efficient template preparation. We are currently investigating the stabilizing properties of alternative carriers such as linear acrylamide to avoid this currently theoretical problem in the future. Finally and more generally, RNA transcripts or cloned viral DNA sequences are available from a relatively narrow range of viruses, typically those that possess relatively small, non‐segmented genomes, and for which full‐length cDNA clones representative of currently circulating strains are available. Although RNA transcripts for EVs and HPeV used in the current study can be readily derived from cloned sequences of a wide range of contemporary circulating virus strains, development of equivalent RNA (or DNA)‐based standards would be problematic or impossible for many respiratory and enteric viruses, and for adenoviruses, herpesviruses and other large DNA viruses with genomes that are too large for conventional cloning strategies.

### 4.2 Analytical sensitivity of EV and HPeV detection assays

As described, the transcripts enabled assay sensitivity to be determined in absolute RNA copy numbers, with almost all assays able to detect 1000 copies of RNA transcripts but with more variable detection of 10 RNA copy controls for EVs and HPeV. There are no current statutory guidelines for EV or HPeV detection sensitivity for diagnostic assays although previous evaluations of widely used EV PCRs found detection limits of 10 to 50 copies for EV and HPeV.^17^ This corresponds to 50 to 200 RNA copies/mL using standard 200 μL extraction volumes of CSF. In the current study, positive results from the low copy number EV or HPeV controls equated to an analytical sensitivity of greater than 50 EV/HPeV RNA genomes/mL for a standard 200 µL CSF extraction volumes. Almost all reference laboratories achieved this sensitivity using in‐house PCR methods, but the 10 copy control was frequently negative in testing with commercial assays. In the case of the biofire film array assay, the mean limit of detection for the 4 EV species was greater than 2000‐4000 RNA copies/mL, with an even lower sensitivity for HPeV3 (Table 2). These findings are consistent with previous evaluations of the Biofire film assay, with reported limits of detection for EV detection of >500 RNA copies/mL,^18^ and some reduction in rates of detection of (unquantified) EV‐positive clinical samples compared to conventional diagnostic assays.^19^, ^20^, ^21^

Viral loads in CSF are low, often at the limit of assay sensitivity of PCR, so variability in assay sensitivity could substantially influence diagnostic target detection rates in diagnostic samples. The development of guidelines for assay sensitivity for EV and HPeV RNA detection in CSF would be of considerable value in the future quality control of these assays. The legislation for the use of CE‐marked tests is driving the replacement of in‐house assays with commercial tests, many of which offer syndromic testing with multiplexed detection of a large range of viruses and bacteria. Although such assays often have operational advantages as point‐of‐care tests in emergency rooms requiring rapid results, the effects of their potentially reduced sensitivity on their clinical utility need to be evaluated. Validation of their performance can be challenging for multiple analytes and existing studies typically do not include samples with defined viral loads or identified EV species or serotypes, nor investigation of samples that have failed detection.^18^, ^19^, ^20^, ^21^ In the future, validation of such assays with calibrated RNA controls is of particular value for evaluation of their performance, particularly when the availability of control material derived from traditional virus isolation methods becomes increasingly restricted.

### 4.3 Virus typing

In the current study, EV and HPeV typing were performed in both reference and diagnostic laboratories (Figure 4). Although all results reported the correct EV‐type identification and most laboratories successfully typed the high concentration (1000 copies in 5 µL) transcripts, fewer than 50% of either reference or diagnostic laboratories could successfully amplify and sequence the low concentration controls. The observed restriction in assay sensitivity underpins the importance of obtaining multiple samples including blood, respiratory samples and feces for EV diagnostic and typing assays where viral loads are higher during acute infections.^22^, ^23^, ^24^ It is also time to consider how EV and HPeV typing data can be centrally collected and analyzed at the time when increasing numbers of diagnostic laboratories are starting to introduce typing within the hospital premises.^7^, ^16^

In conclusion, effective EV and HPeV detection and type identification are integral to clinical management, public health surveillance and outbreak preparedness for emerging strains. However, their genetic diversity, and often low viral loads in diagnostic specimens places stringent demands on the analytical sensitivity and breadth of detection and typing assays. RNA transcripts provide the means to independently evaluate these aspects of their performance. In the future, they can provide objective and fixed standards needed for a more critical assessment of the effectiveness of the numerous, newly developed and currently largely unevaluated testing platforms for syndromic testing. We would be delighted to provide EV, HPeV and further RNA transcript controls for a wider range of viruses to laboratories for QA purposes in the future.

## Supporting information

Supporting information
