# Supplementary material for: A European multicentre evaluation of detection and typing methods for human enteroviruses and parechoviruses using RNA transcripts
Source: J Med Virol. 2020 Jan 17;92(8):1065–74. doi: 10.1002/jmv.25659 (PMC7496258; doi:10.1002/jmv.25659)
Supplement: Supplementary file 1 — Supporting information [file JMV-92-1065-s001.pdf]

SUPPLEMENTARY DATA

SUPPLEMENTARY TABLE S1

Sequences of primers and probes targeting the 5’UTR used in RT-PCR

**Target**	**Primer**	**Sequence and label (listed 5’□ 3’)**
EV	EQ-1	ACATGGTGTGAAGAGTCTATTGAGCT
	EQ-2	CCAAAGTAGTCGGTTCCGC
	EP	6-FAM-TCCGGCCCCTGAATGCGGCTAAT-TAMRA
HRV	235HRV	GACARGGTGTGAAGSYC
	236HRV	CAAAGTAGTYGGTCCCATCC
	522HRV-TQ-FAM	FAM-TCCTCCGGCCCCTGAATGYGGCTAA-BHQ-1
HPeV	F31	CTGGGGCCAAAAGCCA
	K30	GGTACCTTCTGGGCATCCTTC
	HPeV-WT-MGB	6-FAM-AAACACTAGTTGTA(A/T)GGCCC-NFQ

SUPPLEMENTARY TABLE S2

Laboratories enrolled in the study

		Result sets reported	
**Lab type**	**Total**	**Detection[1]**	**Typing**
Reference	12	22 (19 + 3)	25
Diagnostic	27	41 (29 + 12)	12

[1]Sub-totals of in-house and commercial assays respectively indicated in parentheses; all typing assays used in-house methods.

SUPPLEMENTARY FIGURE 1

Relative quantitation of 1000 and 10 RNA copies /µl transcript dilutions

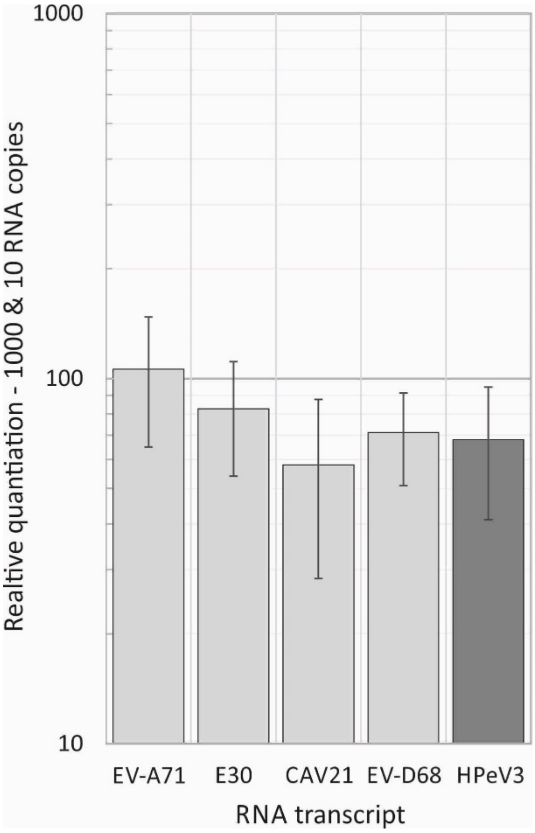

Ratios of Ct values obtained from testing the 1000 and 10 RNA copies / 5ul transcripts of elative detection, expressed as quantitation differences (y-axis; expected value 100). Bars show geometric mean ratios for Ct values for the two dilutions reported by participating laboratories where both dilutions were positive in the screening assay (23 of the 34 result sets).

SUPPLEMENTARY METHODS

EV and HPeV detection and typing methods used by participating laboratories

L1.

EV detection was done using in-house method with the following primers and probe: EV08-1 5'- GGT GCG AAG AGT CTA TTG AGC -3'; EV08-2 5'- CAC CCA AAG TAG TCG GTT CC -3'; Entero-probe 6-FAM 5'- CCG GCC CCT GAA TG -3' MGBNFQ. The PCR mixture contained: 5 µl of RNA template, 1 µM of forward and reverse primer (0.8 µl of 25 µM stock solution), 0.2 µM of probe (0.8 µl of 5 µM stock solution), 5 µl of 4X TaqPath Mastermix, and RNase-free water to a total volume of 20 µl. The PCR cycling profile was: 1x 2 min at 25°C, 15 min at 50°C, 2 min at 95°C (cDNA synthesis); 45x: 3 s at 95°C, 30 s at 56°C (PCR-amplification). EV typing was done using primers by[1], as described in[2].

L3

Data set 1: realtime-RT-PCR Enterovirus. RNA was extracted using the QIAamp Viral RNA mini kit, from 140µl input to 60µl extraction volume. 5µl template added in a 25µl total master mix volume. The method is partially published from Watkins-Riedel T et al.[3]. The thermal cycling parameters are 50C for 30 min, 95C for 15 min, then 45 cycles of 95C for 15 sec and 63C for 1 min.

Data set 2: Entero/EVD68 real-time multiplex PCR was performed as previously described:[4]

Data set 3: Sequencing. RNA extracted using the QIAamp Viral RNA mini kit, from 140µl input to 60µl extraction volume. RNA was amplified using primers SO224/SO222 for PCR1 and AN89/AN88 for PCR2. Amplification and sequencing was based the following protocol:

http://www.euro.who.int/__data/assets/pdf_file/0020/272810/EnterovirusSurveillanceGuidelines.pdf

Data set 4: CoxA16 and EV71 real-time multiplex PCR. RNA was extracted using the QIAamp Viral RNA mini kit, from 140µl input to 60µl extraction volume. 5µl template added in a 25µl total master mix volume. The thermal cycling parameters are 50C for 20 min, 95C for 15 min, then 45 cycles of 95C for 15 sec and 55C for 1 min. The method was based upon the following published protocol[5]

L4

The details of the RT-qPCR assay for routine diagnosis of enteroviruses have been described previously[6]. For the detection of human parechoviruses an in-house one-step RT-qPCR assay was

employed. Viral RNA was extracted from 400µl of clinical specimen using the iPrep PureLink Virus kit according to manufacturer's instructions and eluted in 100µl. Ten microliter of the extracted viral RNA were then used as template in a one-step RT-PCR assay using specific primers (HPeV_F: CAA ACA CTA GTT GTA AGG CCC; HPeV_R: GCC CCA GAT CAG ATC CAY AGT G) and probe (HPeV_P: CCT RCG GGT ACC TTC TGG GCA TCC TTC) targeting a conserved region in the 5'NC region. The reaction was carried out in a total volume of 25 µL containing 12.5µl of a 2x AgPath-ID One-Step qRT-PCR MasterMix (ThermoFisher Scientific), 12.5 pmol of each primer and 5 pmol probe and subjected to the following protocol: an RT step at 48°C for 30min, followed by 95°C for 10 min and subsequent 45 cycles of 95°C for 15s and 60°C for 60s.

L5

In-house RT-PCR targeting the 5'UTR was performed using the following primers: Forward primer - CAT GGT GYG AAG AGT CTA TTG AGC TA; Reverse primer - CCA AAG TAG TCG GTT CCG C and probe FAM-TCC GGC CCC TGA ATG C-MGB. PCR amplification used the the SuperScript™ III One-Step RT-PCR System with Platinum™ Taq DNA Polymerase kit from Thermo Fisher with the following thermal cycle parameters: 50°C for 15 mins 1 cycle; 95°C for 2 mins 1 cycle; 95°C for 15 secs; 56°C for 34 secs; 40 cycles; 72°C for 10 secs.

Parechoviruses were detected by a previously described screening assay[7]

Enteroviruses were typed as previously described[8]. Parechoviruses were typed as previously described[9].

L6

Samples were tested by real time PCR as previously described[10]. For enteroviruses typing In-house One step- reverse transcriptase PanEV PCR targeting whole capsid followed by deep sequencing using Miseq platform and data analysis on Geneious software version 10 was carried out according to our previously published methodology[11]. For enterovirus typing using Sanger sequencing nested PCR approach was used targeting VP1 region[12] using primers 224 and 222 as described previously[8].

L7

Samples were tested by a previously described method[13]

L9

Transcripts were tested by In-house PCR as previously described[14], and by the Meningitis Viral 2 ELITe MGB® Panel (ELITechGroup Molecular Diagnostics, Puteaux, France). RNA transcripts were processed according to the manufacturer's protocol on InGenius, an automated cassette based

sample-to-results solution combining a universal extraction and independently controlled Real-time PCR thermal cycler (ELITechGroup Molecular Diagnostics, Puteaux, France). The InGenius instrument was supplied with amplification Internal Control (IC), the Meningitis Viral (MV) 2 ELITe MGB amplification Master mix, and extraction and amplification cassette consumables provided by the manufacturer (ELITechGroup Molecular Diagnostics, Puteaux, France). Results interpretation was performed according to the instruction manual of the MV 2 ELITe MGB® assay.

L10

EV /PeV detection was performed using the RealCycler EVPA from PROGENIE MOLECULAR, Lot PH2507C, as specified by the manufacturer. Typing was done by amplification and sequencing of the VP1 gene as described in the "Enterovirus surveillance guidelines". CDC. WHO 2015.

L11

RNA extraction was extracted by QIAmp Viral RNA Mini Kit into an elution volume of 60 µl. Samples were iitally tested using a screening PCR (5'NCR region, test volume 2 µl[15]. All PCR products were sequenced and based on the resulting EV species tested with species-specific VP1 PCR assays - EV-A:[16], EV-B: [17]; EV-C:[18]; EV-D:[19].

L12

Samples were screened for enterovirus by in-house PCR in the 5'UTR using the following primers: EV-1s (23nt): 5' GGT GCG AAG AGT CTA TTG AGC TA 3' and EV-1as (19nt): 5' ACC CAA AGT AGT CGG TTC C 3' and EVprobe (24nt): 5' 6FAM-TGA ATG CGG CTA ATC CTA ACT GCG-DB 3'. One-Step-RT-Real-Time-PCR was perfomed on the 2.0 LightCycler system in 20 µl Lightcycler-capillaries using the QuantiFast® Multiplex RT-PCR + R Kit (Qiagen), primers at a final concentration of 0,4 µM each, probe at a final concentration of 0,2 µM and RNAase Inhibitor (Fermentas) at a final concentration of 20 Units. Input-RNA was 5,0 µl, and 1,7 µl of water was added to reach a final reaction volume of 20 µl. RT was done at 50°C for 20 min, followed by 5 min denaturation at 95°C and 45 amplification cycles with denaturation for 15 sec at 95°C, annealing for 10 sec at 56°C and elongation for 30 sec at 60°C. Amplification was monitored after each elongation step at 530 nm.

Parechovirus detection used a previously published method[20]

Samples were further tested for entero-rhinoviruses by NxTAG Resp. Pathogen Panel (Luminex, Lot# I051C0449-NRPB-A-OT) according to manufacturer's instructions.

Samples were typed by amplification and sequencing of the enterovirus VP1 region or VP2/VP4 for enterovirus and rhinovirus positive samples as previously described[8]. Parechovirus typing was performed as previously described[20]

L13

All samples were extracted from aliquots of 200 µl using magLEAD 12gC (PSS) with an elution volume of 100 µl. A first PCR reaction was followed by a semi-nested PCR, primers and protocols were used as previously described[21], with modifications: The cycling programme for PCR1 was 1 cycle of 45° for 30 min and 94° for 2 min followed by 40 cycles of 94° for 15 s, 45° for 45s, and 68° for 1 min, and a final step of 68° for 5 min. For PCR2, a cycle of 95° for 10 min was followed by 30 cycles of 95° for 30 s, 54° for 45 s, and 72° for 45 s, and finally an elongation step of 72° for 7 min. Both PCRs were performed using 0.3 µM of each primer in a final reaction volume of 25 µl. For PCR1 and PCR2, the SuperScript III One-Step RT-PCR System with Platinum Taq DNA Polymerase kit (ThermoFisher Scientific) and the AmpliTaq Gold 360 kit (ThermoFischer Scientific) were used, with 5 µl and 1 µl of template, respectively.

Typing was performed by amplicon sequencing using the same primers as used in the semi-nested PCR. Sequencing reactions were performed on an Applied Biosystems genetic analyzer 3500 XL (Applied Biosystems) using the BigDye Terminator v3.1 Cycle Sequencing Kit (Applied Biosystems).

L15

Parechovirus primers and probes, as described in[22], Parechof31, (5′-CTGGGGCCAAAAGCCA-3′):[23], ParK30, (5′-GGTACCTTCTGGGCATCCTTC-3′):[24] and HPEV-MGB-Probe, 6′FAM 5′-AAACACTAGTTGTA(A/C/T)GGCCC–3′MGB:[22]. Enterovirus primers and probes were as described in[25]. The multiplex-RT-qPCR for parecho and enteroviruses was carried out using Invitrogen SuperScript III Platinum One-Step qRT-PCR (Thermo Fischer) with primers of Parechof31 (400nM), ParK30 (400nM), EQ1 (400nM), EQ2 (400nM), and probes of HPEV-MGB (FAM; 200nM) and Entero (VIC; 50nM) (Smura et al., manuscript in preparation). The PCR program consisted of reverse transcription step of 15min at 50°C followed by initial denaturation of 2min 95°C, and 45 cycles of 15 seconds 95°C and 50 seconds 60°C. 7ul of template was used.

L16

To follow

L17

EV /PeV detection and typing were performed as previously described[9]

L18

RNA transcripts were not re-extracted prior to testing. Screening of a 10µl test volume was performed on the semi-automated High-Plex 24 System (AusDiagnostics Pty. Ltd.) using two multiplex tandem PCR (MT-PCR) assays: Respiratory Viruses 16-well (ref. 20602) version 16, and Viral 8-well (ref. 27093) version 01, as per manufacturer's instruction. Results were analysed using MT Analysis software (version 1.7.2) (AusDiagnostics Pty. Ltd.) and expressed as arbitrary units, as calculated relative to the internal control.

L19

Ten µl volumes of transcripts were tested directly without extraction using the AusDiagnostics Viral (8-well) assay (catalogue number 27093, version 3). Amplification and analysis performed using the AusDiagnostics Easy Plex processor and AusDiagnostics High-Plex 24 system.

L20

Duplicate real-time PCR reactions were set up manually with 8 µl of template in a total volume of 15 µl, using the Taqman Fast Virus 1-step RT-PCR reaction mastermix (Applied Biosystems). Enterovirus and parechovirus reactions were set up in separate parallel wells. Amplification and detection were performed on a 384-well Roche LightCycler 480 II using the following standardised cycling conditions for both targets: 50˚C for 5 minutes, 95˚C for 20 seconds, then 45 cycles of 95˚C for 3 seconds and 60˚C for 30 seconds. Crossing point values were generated by second derivative maximum analysis.

L21

All samples where extracted using the PSS MagLEAD 12gC automated nucleic acid extraction system (Precision System Science). Test volume for all samples was 200ul, elution volume 50ul. Synthesis of cDNA was carried out in a 20-µl reaction mixture containing 5 µl RNA using SuperScript III Reverse Transcriptase (Thermo Fischer Scientific), according to manufacturer's instructions. Following incubation at 25°C (5 min), 50°C (60 min) and 70°C (15 min), 5 µl of the reaction mixture was then used for the first PCR1 (VP1-PCR). PCR1 was performed in a final volume of 50 µl using primers 222 and 224[8] and following amplification (40 cycles) with 95°C (20 sec), 53°C (20 sec) and 72°C (45 sec); 72°C (2 min), 5 µl of the reaction mixture was then used for the second PCR (nested PCR) using primers AN88 and AN89[8]. Amplification was carried out at (40 cycles) with 95°C (20 sec), 65°C (20 sec) and 72°C (45 sec); 72°C (2 min) using the KAPA2G Robust DNA Polymerase (5 U/µL) för both PCRs. Initial denaturation was achieved by incubation at 95°C for 60 sec prior to the amplification

cycles. Reaction products were separated and visualized on 2% agarose gel containing ethidium bromide (E-Gel® 2% double comb with Ethidium Bromide, Invitrogen). For sequencing, PCR products are purified using ExoSAP-IT (Thermo Fischer Scientific) and the BigDye Terminator v3.1 Cycle Sequencing Kit (Invitrogen) was used for Sanger sequencing. For sequence analysis the CLC workbench and the RIVM´s Enterovirus typing tool (https://www.rivm.nl/mpf/typingtool/enterovirus/) were used.

L22

Investigation performed using Fast Track Diagnostics FTD Viral Meningitis Kit (FTD-13-64) Lot no. VM19-64-02, expiry 2020-05. 15ul MasterMix plus 10ul template run on Roche LightCycler 480 II. Cycling conditions as per manufacturer: 50C 15 minutes Hold, 94C 1 minute Hold, 94C 8 secs 60C 60 secs (45 cycles).

L24

Samples were extracted by NucliSens EasyMag automated system (BioMérieux), and EV and HPeV sequences amplified as previously described[26].

L25

Detection of enterovirus and parechovirus was performed by commercial real-time RT-PCR assays (Allplex Respiratory Panel 2 Assay, Seegene, and, Real Cycler EVPA, Progenie), according to the manufacturer's instructions. Genetic characterisation of enteroviruses was performed based on VP1 sequencing as recommended by the WHO, with minor modifications.

L26

A one-step real-time multiplex RT-PCR assay was carried out to simultaneously detect EV and HPeV genome using specific primer/probe sets targeting the 5′ untranslated region (5′ UTR)[27]. The reaction mixture contained 1 µM of each EV primer, 0.4 µM of each HPeV primer and 0.2 µM of each probe and was carried out with the following thermal profile: 50 °C×30 min, 95 °C×15 min, and 50 cycles at 95 °C×15 s, 58 °C×30 s, and 72 °C×10 s. The real-time RT-PCR assays were set up using 5 µl of RNA in a final reaction volume of 25 µl of AgPath-ID one-step RT-PCR reagents (Thermo Fisher Scientific, USA).

The EV-positive samples were then tested using a one-step real-time RT-PCR assay specific for 5′ UTR of EV-D68[28]; The reaction mixture contained 0.6 µM of EVD68 primer and 0.2 µM of the two EVD68 probes and were run as follows: 50 °C×30 min, 95 °C×20 s and 45 cycles of 95 °C×20 s, 50 °C×30 s and 60 °C×45 s. The real-time RT-PCR assays were set up using 10 µl of RNA in a final reaction volume of 15 µl of AgPath-ID one-step RT-PCR reagents (Thermo Fisher Scientific, USA).

All Real-time assays were performed in a PlusOne real-time PCR system (Thermo Fisher Scientific, USA). A sample was considered positive when its Ct value was <40.

The molecular characterization of EV and HPeV was carried out using specific primers targeting the gene encoding for the viral protein (VP) 1 of EV (nt. 2602–2977)[8] and the VP3/VP1 junction region of HPeV (nt. 2159–2458)[29]. Amplicons were purified using a commercial purification kit (NucleoSpin Gel and PCR clean-up kit, Macherey-Nagel, Germany) and sequenced according to the Sanger method.

L27

RNA was extracted using a NucliSens easyMag, bioMeriéux with extraction volumes 200µL and elution volumes 55µL. Mastermix used for the 1-step real-time PCR for detection of enterovirus was QuantiTect Probe RT-PCR kit, Qiagen and the instrument for real-time is CFX96TM , Biorad (30 min 50°C ,15 min 95°C followed by 40 cycles 95°C 15 sec og 60°C 30 sec). 5µl eluate was added to 15µl reaction mix.

For the detection of parechovirus and rhinovirus a 2-step real-time PCR was performed. 10µl eluate was used in the reverse transcription step with random primer, Promega and M-MLV Reverse Transcriptase, Invitrogen. Further, 5µl cDNA and 15µl reactionmix was used for the real-time PCR. The mastermix was Custom Multiplex PCR SuperMix, Quanta Biosciences, and the instrument for real-time was a CFX96TM, Biorad (45°C 5min, 95°C 3min and 40 cycles 95°C 5sec, 55°C 30sec).

Virus typing was performed by PCR and Sanger sequencing using previously described primers for primers for enterovirus[8], parechovirus[30] and rhinovirus[1]. Sanger sequencing is performed with BigDye Terminator v.3.1, Applied Biosystems and sequences are analyzed on ABI Hitachi 3130XL Genetic Analyzer, Applied Biosystems

L29

EV genome detection. Viral RNA was extracted from 200 µl of sample on the NucliSens EasyMag automated system (bioMérieux) with the specific B protocol (elution volume of 50µl). Real time RT-PCR was then performed with the kit Enterovirus R-gene (bioMérieux) with 10 µl of the RNA extract.

Parechovirus genome detection. Viral RNA was extracted from 200 µl of sample on the NucliSens EasyMag automated system (bioMérieux) with the specific B protocol (elution volume of 50µl). Real time RT-PCR was then performed with the kit parechovirus r-gene (bioMérieux) with 10 µl of the RNA extract.

Typing. EV-B and EV-A were typed as described previously[31]. Amplification of the rhinovirus 1A-1B region uses primers described by Savolainen et al[32] in a one-step RT-PCR and if negative, we

performed a second PCR from the PCR products of the first amplification using the primers described by Linsuwanon[33].

L30

Multiplex real-time RT-PCR assays. All multiplex real-time PCR assays were performed in a Roche Lighcycler 480II apparatus. All assays included a primer/probe set targeting the human Ribonucleoprotein P (RNP) gene as internal control for the quality of the sample, extraction and amplification using previously described primers and probes[34]. Laboratory procedures to prevent PCR contamination were strictly followed and positive controls (known positives; purified viral nucleic acids -Vircell, Spain-; and titered HEV virus culture supernatants -a gift from María Cabrerizo, ISCIII, Spain-) and negative controls (UTM and/or nucleic acid) were included in each round.

Rhinovirus/enterovirus detection assay. For respiratory virus detection, our lab performs four different screening RT-PCR assays, using 5 ul of the eluted nucleic acid for each assay, 10 ul of 2X Quanta qScript XLT PCR Master mix + enzyme mix (Quanta Biosciences) and 20U of RNAsin (Promega) in a final reaction volume of 20 ul. For rhino/enterovirus detection, one of our four multiplex screening assays detects both human rhinovirus (HRV) and human enteroviruses (HEV) using probes from the 5'UTR region[35].

HEV / HRV assay. Differentiation of HEVs from HRVs was performed using a common primer set for the human rhinovirus/enterovirus 5'UTR gene, and differential LNA probes for HEV (Cy5) and rhinoviruses (HEX)[28], using the same reaction set-up as above.

HEV-D68 type-specific assay. Detection of HEV-D68 was performed using a primer/probe (VIC) set specific for the HEV-D68 5'UTR gene-[28] using the same reaction set-up as above.

L31

Samples were screened for EV and HPeV by Biofire Film array ME panel v1.4 according to the manufactuer's instructions.

L34

Detection of EV/HPeV by in-house is performed as described previously[36]. Enteroviruses were typed as described[21,25]. HPewV sequences were types as previously described[37].

L35, L46

EV and HPeV were detected by RT PCR as previously described[7,38]. EVs were typing by sequencing of the VP1 gene as previously described[8].

L36

EV detection was done using in-house method targeting a highly conserved area of the 5’ untranslated region with the following primers and probe: EV08-1 GGTGCGAAGAGTCTATTGAGC, EV08-2 CACCCAAAGTAGTCGGTTCC and MGB (minor groove binder) probe Fam-CCGGCCCCTGAATG as previously described[36].

L37

In House Testing – Transcripts were tested directly without prior extraction. Random primed cDNA was prepared using Promega MMLV reverse transcriptase (Cat No. M1701) in 25µL reactions containing 50mM Tris-HCl (pH 8.3), 75mM KCl, 3mM MgCl2, 10mM DTT, 5 ng/µL random hexamers (Sigma Aldrich, Cat No. 11034731001), 200 units MMLV reverse transcriptase and 18µL of transcript and incubated at 37°C for 30 minutes followed by heat inactivation at 95°C for 10 minutes. 5µL of prepared cDNA was added to real time PCR reactions containing 1x Applied Biosystems Fast Universal PCR Master Mix (Thermo Fisher Cat No. 4352042), 300nM each primer, 100nM probe in a final reaction volume of 20µL. PCR reactions were carried out using the Applied Biosystems QuantstudioTM 7 with cycling parameters 1 cycle of 50°C 120s and 95°C 20s followed by 50 cycles of 95°C 3s and 60°C 60s.

Altona EV/RhV Assay – Transcripts were tested directly without prior extraction according to the manufacturer’s protocol (Altona Diagnostics RealStar® Enterovirus RT-PCR Kit v1.0, Cat No. 571013). 5µL of each transcript was added to 26µL of reaction mix comprising 5µL Master A, 15µL Master B, 1µL Internal Control and 5µL molecular grade water. PCR reactions were carried out using the Applied Biosystems QuantstudioTM 7 with cycling parameters 1 cycle of 55°C 20 minutes and 95°C 2 minutes followed by 45 cycles of 95°C 15s, 55°C 45s and 72°C 15s.

L38

Viral RNA was extracted using the QIAamp viral RNA mini kit (QIAGEN, Lübeck, Germany) according to the manufacturer’s instructions.

PCR Screening assay #1. A commercial multiplex real-time PCR assay (FTD Neuro 9 multiplex real-time PCR Kit, Fast Track Diagnostics, Siemens Healthineers, Germany) was used. Ten ul of RNA was used in a total reaction volume of 25 ul.

PCR Screening assay #2. Transcripts were tested using an in-house PCR as previously described[39].

Enterovirus typing. a nested RT-PCR which amplifies the VP1 coding region was applied using the Qiagen One Step RT-PCR kit and primer sets and cycling conditions as described by Nix *et al.* [8]. The

total volume of the PCR reactions was 25 ul. Five ul cDNA was used for the first round, while 1ul of the PCR product was used for the second round.

L39

A commercial multiplex real-time PCR assay, Seegene Allplex test kit and an in-house PCR method based on[40] was used for enterovirus / rhinovirus screening. Parechoviruses were detected by in-house as previously described[41] using primers listed in[42].

L40

Enteroviruses were detected using in-house one-step real-time reverse transcription PCRs (RT-RT-PCR) adopted from published literature.

Method 1: in the RT-RT-PCR reaction, primers and probe set was used as published by Dierssen *et al*[25]. The sequences as follows: EV-F (5'-3'): ACATGGTGTGAAGAGTCTATTGAGCT; EV-R (5'-3'): CCAAAGTAGTCGGTTCCGC; EV-probe (5'-3'): TCCGGCCCCTGAATGCGGCTAAT. The qScript XLT 1-Step RT-qPCR ToughMix (Quanta Biosciences, cat.no. 95132-100) was used for master mix preparation according to manufacturer's instructions (master mix volume = 12 µL). The volume of RNA transcript in the reaction was 3 µL. The total volume of the reaction was therefore 15 µL. Temperature profile was as follows: (50˚C–10min) → (95˚C–1min) and followed by 45 cycles (95˚C–10s → 60˚C–45s). A RotorGen Q machine was used.

Method 2: in the RT-RT-PCR reaction, primers and probe set was used as published by Cui et al.[43]. The sequences as follows: EV(YG)-F (5'-3'): GGCTGCGYTGGCGGCC; EV(YG)-R (5'-3'): CCAAAGTAGTCGGTTCCGC; EVTY(YG)-probe (5'-3'): CTCCGGCCCCTGAATGCGG. The AgPath-ID One-Step RT-PCR (ThermoFisher Scientific, cat.no. AM1005) was used for master mix preparation according to manufacturer's instructions (master mix volume = 20 µL). The volume of RNA transcript in the reaction was 5 µL. The total volume of the reaction was therefore 25 µL. Temperature profile was as follows: (45˚C–10min) → (95˚C–10min) and followed by 45 cycles (95˚C–5s → 55˚C–35s). A RotorGen Q machine was used.

Parechoviruses were detected using in-house one-step real-time reverse transcription PCRs (RT-Rt-PCR) adopted from published literature. In the RT-RT-PCR reaction, primers and probe set was used as published by Nix et al.[38] The sequences as follows: HPev-F (5'-3'): GTAACASWWGCCTCTGGGSCCAAAAG; HPeV-R (5'-3'): GGCCCCWGRTCAGATCCAYAGT; HPeV-probe (5'-3'): CCTRYGGGTACCTYCWGGGCATCCTTC. The qScript XLT 1-Step RT-qPCR ToughMix (Quanta Biosciences, cat.no. 95132-100) was used for master mix preparation according to manufacturer's instructions (master mix volume = 15 µL). The volume of RNA trasnscript in the reaction was 5 µL.

The total volume of the reaction was therefore 20 µL. Temperature profile was as follows: (50˚C–10min) → (95˚C–1min) and followed by 45 cycles (95˚C–10s → 55˚C–45s). A RotorGen Q machine was used.

L42

Samples are extracted on the Qiasymphony instrument (Qiagen) using the DSP virus/pathogen kit (Qiagen) with carrier RNA and an internal RNA-virus control (PDV) added to every sample. Qualitative, one-step RT-PCR is performed on the ABI 7500 instrument (Thermofisher) using Quantifast multiplex PCR + R RT mastermix (Qiagen). Separate assays are perfomed for EV and HPeV targets, both are multiplexed with the PDV assay.

Primer and probe sequences for EV are based on a previous publication[44], but with a modified probe sequence that is truncated and has the minor groove binding (MGB) modification (EV-UTR-F: ACA TGG TGC GAA GAG TCT ATT GAG CT; EV-UTR –R: GAA ACA CGG ACA CCC AAA GTA GTC G; EV-UTR –probe: FAM-TCC GGC CCC TGA AT-MGB). Primer and probe sequences for parechovirus were designed in-house and comprised Parechovirus-F: CGA AGG ATG CCC AGA AGG T; Parechovirus-R: GCC CCA GAT CAG ATC CAT AGT G and Parechovirus-probe: VIC-CCC GTA GGT AAC AAG TG - MGB. Internal control primers were PDV-F: GCG GGT GCC TTT TAC AAG AAC; PDV-R: CAG AAT AAG CAA AAT TGA TAG GAA CCA T and PDV-Probe: CY5-TCT TTC CTC AAC CTC GTC CGT CAC AAG T-BHQ2.

L43

For EV and HPeV detection, 9 µl of RNA transcript was added to real-time PCR master mix (Invitrogen EXPRESS One-Step Superscript® qRT-PCR Universal 2x master mix, Invitrogen EXPRESS One-Step Superscript® qRT-PCR Universal RT enzyme mix and primer/probe mix) up to a total volume of 25 µL. The EV primers and probe were as previously designed for one-step real-time RT-PCR[25]. The HPeV primers and probe were directly adopted from[42]. The Real-time PCR cycling conditions were as follows: 50°C for 15 min and 95°C for 20 min followed by 45 cycles of 95°C for 3 sec, 55°C for 30 sec and 72°C for 10 sec performed on the 7500 Real-time PCR system (Applied Biosystems, Warrington, UK).

Positive samples were typed by combined first round RT and PCR followed by a second amplification reaction was performed for amplifying the EV VP1 and VP4 regions and HPeV VP3/VP1 region before sequencing the amplified DNA. For both the VP1 and VP4 outer EV PCRs, 6 µl of RNA transcript was added to RT-PCR master mix (Invitrogen Superscript® III One-Step RT-PCR 2x Master Mix, Invitrogen Superscript® III RT / Platinum Taq HiFi enzyme mix and primers) for a reaction volume of 20 µl. The EV primers were previously described[1,17].

For the outer HPeV PCR, 6 µl of RNA transcript was added to RT-PCR master mix (Invitrogen EXPRESS One-Step Superscript® qRT-PCR Universal 2x master mix, Invitrogen EXPRESS One-Step Superscript® qRT-PCR Universal RT enzyme mix and primers) for a reaction volume of 20 µl. The HPeV primers were previously described in[29]. Cycling conditions for EV VP1 outer PCR: 43°C for 1 hour followed by 20 cycles of 53°C for 1 min and 55°C 1 min, 70°C for 15 min, 94°C for 2 min, followed by 40 cycles of 94°C for 30 sec, 45°C for 30 sec and 68°C for 1 min 45 sec, 68°C for 5 min. Cycling conditions for EV VP4 and HPEV outer PCR: 43°C for 1 hour followed by 20 cycles of 53°C for 1 min and 55°C 1 min, 70°C for 15 min, 94°C for 2 min, followed by 40 cycles of 94°C for 30 sec, 50°C for 30 sec and 68°C for 1 min 45 sec, 68°C for 5 min.

For the inner PCR for all assays, 1 µl of 1st round product was added to PCR master mix (Qiagen HotStarTaq 10x Buffer, 3mM dNTPs, Qiagen HotStarTaq enzyme and primers) for a reaction volume of 20 µl. Cycling conditions for EV VP1 inner PCR: 95°C 15 min followed by 30 cycles of 94°C for 18 sec, 48°C for 21 sec and 72°C for 1 min 30 sec, 72°C for 5 min. Cycling conditions for EV VP4 and HPEV inner PCR: 95°C 15 min followed by 30 cycles of 94°C for 18 sec, 50°C for 21 sec and 72°C for 1 min 30 sec, 72°C for 5 min.

Amplified DNA was directly sequenced using BigDye Terminator kit v3.1 (Applied Biosystems, Warrington, United Kingdom) in both directions and analysed on the 3500XL genetic analyser (Applied Biosystems, Warrington, United Kingdom). Type identification was achieved by the online Enterovirus typing tool found at: https://www.rivm.nl/mpf/typingtool/enterovirus/

L44.

Pan-enterovirus detection was carried out using primers AN350 and AN351 and probe AN234[45], 5 µl RNA and qScript™ XLT One-Step RT-qPCR ToughMix® (Quanta Biosciences, Beverly, MA, USA) in a final reaction volume of 20 µl. Cycling conditions were 50°C for 30 minutes, 95°C for 5 minutes followed by 50 cycles of 95°C for 15 seconds, 55°C for 45 seconds and 72°C for 15 seconds. For specific EV-A71 detection, 5 µl RNA, primers and probe from[5] and SuperScriptTM III Platinum® One-Step Quantitative RT-PCR System (Invitrogen, Carlsbad, CA, USA) were used. 50 µl reactions were run in the same cycling conditions as the pan-enterovirus RT-PCR. For EV-D68 detection, 10 µl RNA was first reverse-transcribed with random hexamer primers (Roche, Basel, Switzerland) and RevertAid H Minus Reverse Transcriptase (Thermo Scientific, Waltham, MA, USA) in a 50 µl volume. 5 µl cDNA was amplified using QuantiTect Multiplex PCR Kit (Qiagen, Hilden, Germany) with primers and probe described in[46] in a 25 µl final volume. PCR cycling conditions were 95°C for 15 minutes followed by 50 cycles of 95°C for 30 seconds, 55°C for 30 seconds and 72°C for 30 seconds. Parechovirus detection was carried out using 1 µl RNA, primers and probe[8] and qScript™ XLT One-Step RT-qPCR

ToughMix® (Quanta Biosciences) in a final reaction volume of 20 µl. The cycling was performed at 50°C for 30 minutes, 95°C for 5 minutes followed by 45 cycles of 95°C for 15 seconds, 58°C for 45 seconds and 72°C for 10 seconds. All PCRs were run in the Mx3005P analyzer (Stratagene, La Jolla, CA, USA).

Both enterovirus and parechovirus VP1 typing were performed exactly as described for clinical specimens in (Enterovirus surveillance guidelines. ISBN 978 92 890 5081 4). PCR amplicons were purified using QIAquick PCR Purification Kit (Qiagen, Hilden, Germany). The sequencing reactions with BigDye Terminator cycle sequencing ready reaction kit v3.1 (Life Technologies, Carlsbad, CA, USA) and sequencing with ABI3730 Automatic DNA Sequencer (Life Technologies) were performed by the Institute for Molecular Medicine Finland (FIMM) Sequencing Laboratory, Helsinki, Finland. The electropherograms were analyzed using Sequencher (Gene Codes Corporation, Ann Arbor, MI, USA).

L45

RNA was extracted using QIAsymphony DSP Virus/Pathogen Mini kit (QIAGEN) and eluted in 60 µl. RT-PCR was carried out using AgPath-ID™ One-Step RT-PCR Kit (Life Technologies) (1x RT-PCR buffer, 0.16 µM enterovirus forward primer (5'- TCC-TCC-GGC-CCC-TGA-AT -3'), 0.16µM enterovirus reverse primer (5'- RAT-TGT-CAC-CAT-AAG-CAG-YCA-3'), 0.16µM enterovirus probe (5'-6-FAM- CGG-AAC-CGA-STA-CTT-TG- MGB NFQ -3’), 0.08µM parechovirus forward primer (5'- ACT-AGT-TGT-A(5)G-GCC-CRY-GAA-GG - 3'), 0.08µM parechovirus reverse primer (5'- ATC-AGA-TCC-AYA-GTG-YC(5)-CTT-GTT-ACC-T- 3'), 0.16µM enterovirus probe (5'-JOE-ATG-CCC-AGA-AGG-TAC-CCG-T-BHQ1-3'), 0.08µM MS2 forward primer (5'-TGG-CAC-TAC-CCC-TCT-CCG-TAT-TCA-CG-3'), 0.08µM MS2 reverse primer (5'-GTA-CGG-GCG-ACC-CCA-CGA-TGA-C-3'), 0.08µM MS2 probe (5'-CY3-CAC-ATC-GAT-AGA-TCA-AGG-TGC-CTA-CAA-GC-BHQ-2-3') and 1x RT-PCR Enzyme mix) and 5µl extract in 25µl reaction volume. RT-PCR was carried out on an ABI 7500 under the following cycling conditions were 50°C for 20 minutes, 95°C for 10 minutes followed by 45 cycles of 95°C for 15 seconds, 60°C for 30 seconds acquiring on FAM, JOE and CY3.

L99

HPeV was tested in three samples C1, C2 and C7 with the nested PCR of Harvala et al[29]. All samples were tested with the NCR PCR for enterovirus[47] and rhinovirus[48]. Positive samples were sequenced to detect the species of enterovirus. Depending on that sequence result different primers sets were used. The outer primer set of Leitch et al[17], was used for the amplification of the VP1 region of an

enterovirus A sample. PCR for enterovirus B was performed with the ENTNES inner primer set of Thoelen et al[47]. If the result of the NCR sequence was an enterovirus D, the nested VP4/VP2 primer set of Wisdom et al[1] was used for PCR and sequencing. If the sequencing result of the NCR was EV-C the VP1 was confirmed with an in house PCR, in this case with coxsackievirus A21 primers F (5'-CCA GGT CTG AAT CGT GCC TT-3') and R (5'-CTT GGT TAC GCA CTT CCC CA -3')..

In a 25 µl reaction volume containing 5 µl 5X QIAGEN One-step RT-PCR buffer, 1 µl dNTP mix containing 200µM of each dNTP, 1 µl QIAGEN One-step RT-PCR enzyme mix, 60 pmol of each primers and RNase-free water. The amplification profile involved a reverse transcription step at 50°C for 30 min, followed by PCR activation at 95°C for 15 min, 40 cycles of amplification (94°C, 30 sec; 55°C, 30 sec; 72°C, 1 min) and a final extension of 10 min at 72°C in a ProFlex PCR System (Applied Biosystems). The PCR products were run on a polyacrylamide gel (PAGE), stained with Midori Green direct (Nippon Genetics Europe) for visualizing the PCR products. Purified PCR products were send to Macrogen for sequencing.

References

1. Wisdom A, McWilliam Leitch C, Gaunt E, Harvala H, Simmonds P. Screening respiratory samples for human rhinoviruses (HRV) and enteroviruses: comprehensive VP4/2-typing reveals high incidence and genetic diversity of HRV species C. *Journal of Clinical Microbiology.* 2009;47:3958-3967.
2. Dyrdak R, Rotzen-Ostlund M, Samuelson A, Eriksson M, Albert J. Coexistence of two clades of enterovirus D68 in pediatric Swedish patients in the summer and fall of 2014. *Infectious diseases (London, England).* 2015;47(10):734-738.
3. Watkins-Riedel T, Woegerbauer M, Hollemann D, Hufnagl P. Rapid diagnosis of enterovirus infections by real-time PCR on the LightCycler using the TaqMan format. *Diagn Microbiol Infect Dis.* 2002;42(2):99-105.
4. Bragstad K, Jakobsen K, Rojahn AE, et al. High frequency of enterovirus D68 in children hospitalised with respiratory illness in Norway, autumn 2014. *Influenza and other respiratory viruses.* 2015;9(2):59-63.
5. Zhang S, Wang J, Yan Q, et al. A one-step, triplex, real-time RT-PCR assay for the simultaneous detection of enterovirus 71, coxsackie A16 and pan-enterovirus in a single tube. *Plos One.* 2014;9(7):e102724.

6. Richter J, Tryfonos C, Panagiotou C, Nikolaou E, Koliou M, Christodoulou C. Newly emerging C group enteroviruses may elude diagnosis due to a divergent 5'-UTR. *International journal of infectious diseases : IJID : official publication of the International Society for Infectious Diseases.* 2013;17(12):e1245-1248.
7. Corless CE, Guiver M, Borrow R, et al. Development and evaluation of a 'real-time' RT-PCR for the detection of enterovirus and parechovirus RNA in CSF and throat swab samples. *Journal of Medical Virology.* 2002;67(4):555-562.
8. Nix WA, Oberste MS, Pallansch MA. Sensitive, seminested PCR amplification of VP1 sequences for direct identification of all enterovirus serotypes from original clinical specimens. *Journal of Clinical Microbiology.* 2006;44(8):2698-2704.
9. Cremer J, Morley U, Pas S, et al. Highly sensitive parechovirus CODEHOP PCR amplification of the complete VP1 gene for typing directly from clinical specimens and correct typing based on phylogenetic clustering. *Journal of medical microbiology.* 2019;68(8):1194-1203.
10. Gerloff N, Sun H, Mandelbaum M, et al. Diagnostic Assay Development for Poliovirus Eradication. *J Clin Microbiol.* 2018;56(2).
11. Majumdar M, Martin J. Detection by Direct Next Generation Sequencing Analysis of Emerging Enterovirus D68 and C109 Strains in an Environmental Sample From Scotland. *Frontiers in microbiology.* 2018;9:1956.
12. Majumdar M, Sharif S, Klapsa D, et al. Environmental Surveillance Reveals Complex Enterovirus Circulation Patterns in Human Populations. *Open forum infectious diseases.* 2018;5(10):ofy250.
13. Honkanen H, Oikarinen S, Pakkanen O, et al. Human enterovirus 71 strains in the background population and in hospital patients in Finland. *J Clin Virol.* 2013;56(4):348-353.
14. Piralla A, Girello A, Premoli M, Baldanti F. A new real-time reverse transcription-PCR assay for detection of human enterovirus 68 in respiratory samples. *J Clin Microbiol.* 2015;53(5):1725-1726.
15. Kuryk L, Wieczorek M, Diedrich S, Bottcher S, Witek A, Litwinska B. Genetic analysis of poliovirus strains isolated from sewage in Poland. *J Med Virol.* 2014;86(7):1243-1248.
16. Bottcher S, Obermeier PE, Neubauer K, Diedrich S. Recombinant Enterovirus A71 Subgenogroup C1 Strains, Germany, 2015. *Emerg Infect Dis.* 2016;22(10):1843-1846.
17. Leitch EC, Harvala H, Robertson I, Ubillos I, Templeton K, Simmonds P. Direct identification of human enterovirus serotypes in cerebrospinal fluid by amplification and sequencing of the VP1 region. *JClinVirol.* 2009;44(2):119-124.

18. Osundare FA, Akanbi O.A., Akindele AA, et al. Detection and characterization of Enteroviruses, Cosaviruses and a new Human Parechovirus type in healthy individuals in Osun state, Nigeria, 2016/2017. *Submitted.* 2019.

19. Bottcher S, Prifert C, Weissbrich B, et al. Detection of enterovirus D68 in patients hospitalised in three tertiary university hospitals in Germany, 2013 to 2014. *Euro surveillance : bulletin Europeen sur les maladies transmissibles = European communicable disease bulletin.* 2016;21(19).

20. Pietsch C, Liebert UG. Genetic diversity of human parechoviruses in stool samples, Germany. *Infection, genetics and evolution : journal of molecular epidemiology and evolutionary genetics in infectious diseases.* 2019;68:280-285.

21. Nasri D, Bouslama L, Omar S, et al. Typing of human enterovirus by partial sequencing of VP2. *J Clin Microbiol.* 2007;45(8):2370-2379.

22. Jaaskelainen AJ, Kolehmainen P, Kallio-Kokko H, et al. First two cases of neonatal human parechovirus 4 infection with manifestation of suspected sepsis, Finland. *J Clin Virol.* 2013;58(1):328-330.

23. Benschop K, Minnaar RP, Koen G, et al. Detection of enterovirus and human parechovirus genotypes from clinical stiool samples; PCR and direct molecular typing, culture characteristics and serotyping. *Journal of Clinical Microbiology.* 2010;(in press).

24. Oberste MS, Maher K, Pallansch MA. Specific detection of echoviruses 22 and 23 in cell culture supernatants by RT-PCR. *Journal of Medical Virology.* 1999;58(2):178-181.

25. Dierssen U, Rehren F, Henke-Gendo C, Harste G, Heim A. Rapid routine detection of enterovirus RNA in cerebrospinal fluid by a one-step real-time RT-PCR assay. *JClinVirol.* 2008;42(1):58-64.

26. McLeish NJ, Witteveldt J, Clasper L, et al. Development and assay of RNA transcripts of enterovirus species A to D, rhinovirus species a to C, and human parechovirus: assessment of assay sensitivity and specificity of real-time screening and typing methods. *J Clin Microbiol.* 2012;50(9):2910-2917.

27. Bubba L, Pellegrinelli L, Pariani E, Primache V, Amendola A, Binda S. A novel multiplex one-step real-time RT-PCR assay for the simultaneous identification of enterovirus and parechovirus in clinical fecal samples. *Journal of preventive medicine and hygiene.* 2015;56(2):E57-60.

28. Poelman R, Scholvinck EH, Borger R, Niesters HG, van Leer-Buter C. The emergence of enterovirus D68 in a Dutch University Medical Center and the necessity for routinely screening for respiratory viruses. *J Clin Virol.* 2015;62:1-5.

29. Harvala H, Robertson I, McWilliam Leitch EC, et al. Epidemiology and clinical associations of human parechovirus respiratory infections. *Journal of Clinical Microbiology.* 2008;46:3446-3453.

30. Nix WA, Maher K, Pallansch MA, Oberste MS. Parechovirus typing in clinical specimens by nested or semi-nested PCR coupled with sequencing. *J Clin Virol.* 2010;48(3):202-207.

31. Mirand A, le Sage FV, Pereira B, et al. Ambulatory Pediatric Surveillance of Hand, Foot and Mouth Disease as Signal of an Outbreak of Coxsackievirus A6 Infections, France, 2014-2015. *Emerg Infect Dis.* 2016;22(11):1884-1893.

32. Savolainen C, Blomqvist S, Mulders MN, Hovi T. Genetic clustering of all 102 human rhinovirus prototype strains: serotype 87 is close to human enterovirus 70. *Journal of General Virology.* 2002;83(Pt 2):333-340.

33. Linsuwanon P, Payungporn S, Samransamruajkit R, et al. High prevalence of human rhinovirus C infection in Thai children with acute lower respiratory tract disease. *JInfect.* 2009;59(2):115-121.

34. Emery SL, Erdman DD, Bowen MD, et al. Real-time reverse transcription-polymerase chain reaction assay for SARS-associated coronavirus. *Emerg Infect Dis.* 2004;10(2):311-316.

35. Tapparel C, Cordey S, Van Belle S, et al. New molecular detection tools adapted to emerging rhinoviruses and enteroviruses. *J Clin Microbiol.* 2009;47(6):1742-1749.

36. Nielsen AC, Bottiger B, Midgley SE, Nielsen LP. A novel enterovirus and parechovirus multiplex one-step real-time PCR-validation and clinical experience. *J Virol Methods.* 2013;193(2):359-363.

37. Harvala H, Robertson I, McWilliam Leitch C, Chieochansin T, Templeton K, Simmonds P. Aetiological role of human parechovirus type 3 in neonatal sepsis identified by direct typing assay on cerebrospinal fluid. *Journal of Infectious Diseases.* 2009;199(12):1753-1760.

38. Nix WA, Maher K, Johansson ES, et al. Detection of all known parechoviruses by Real Time-PCR. *Journal of Clinical Microbiology.* 2008;46:2519-2524.

39. Takami T, Kawashima H, Takei Y, et al. Usefulness of nested PCR and sequence analysis in a nosocomial outbreak of neonatal enterovirus infection. *J Clin Virol.* 1998;11(1):67-75.

40. Osterback R, Tevaluoto T, Ylinen T, et al. Simultaneous detection and differentiation of human rhino- and enteroviruses in clinical specimens by real-time PCR with locked nucleic Acid probes. *J Clin Microbiol.* 2013;51(12):3960-3967.

41. Linden S, Vuorinen T, Osterback R, Soilu-Hanninen M. Parechovirus infection preceding Guillain-Barre syndrome. *J Neurovirol.* 2012;18(5):434-436.

42. Benschop K, Molenkamp R, van der Ham A, Wolthers K, Beld M. Rapid detection of human parechoviruses in clinical samples by real-time PCR. *JClinVirol.* 2008;41(2):69-74.
43. Cui A, Xu C, Tan X, et al. The development and application of the two real-time RT-PCR assays to detect the pathogen of HFMD. *Plos One.* 2013;8(4):e61451.
44. Cabrerizo M, Diaz-Cerio M, Munoz-Almagro C, et al. Molecular epidemiology of enterovirus and parechovirus infections according to patient age over a 4-year period in Spain. *J Med Virol.* 2017;89(3):435-442.
45. Kilpatrick DR, Yang CF, Ching K, et al. Rapid group-, serotype-, and vaccine strain-specific identification of poliovirus isolates by real-time reverse transcription-PCR using degenerate primers and probes containing deoxyinosine residues. *J Clin Microbiol.* 2009;47(6):1939-1941.
46. Poelman R, Schuffenecker I, Van Leer-Buter C, Josset L, Niesters HG, Lina B. European surveillance for enterovirus D68 during the emerging North-American outbreak in 2014. *J Clin Virol.* 2015;71:1-9.
47. Thoelen I, Lemey P, Van Der Donck I, Beuselinck K, Lindberg AM, Van Ranst M. Molecular typing and epidemiology of enteroviruses identified from an outbreak of aseptic meningitis in Belgium during the summer of 2000. *J Med Virol.* 2003;70(3):420-429.
48. Kiang D, Kalra I, Yagi S, et al. Assay for 5' noncoding region analysis of all human rhinovirus prototype strains. *Journal of Clinical Microbiology.* 2008;46(11):3736-3745.
